# Supplementary material for: How FAIR is metadata for human pluripotent stem cells?
Source: Stem Cell Reports. 2025 Sep 25;20(10):102644. doi: 10.1016/j.stemcr.2025.102644 (PMC12790715; doi:10.1016/j.stemcr.2025.102644)
Supplement: Document S1. Figures S1–S3 and Tables S1 and S3 [file mmc1.pdf]

Stem Cell Reports, Volume *20*

Supplemental Information

How FAIR is metadata for human pluripotent stem cells?

Mengqi Hu, Rachel A. Ankeny, Dan Santos, and Christine A. Wells

Hu et al Stem Cell Reports How FAIR is metadata for human pluripotent stem cells?

Supplementary Files Index

Figure S1. Effect of Time (Publication Year) on Citation Count of Cell Lines. Citations of published cell lines are shown in a dot plot, with the Y-axis representing publication year and the X-axis representing citation count. Citation counts were log-transformed (log10 + 1) to ensure all values are positive. A linear regression line (red) was fitted using Ordinary Least Squares (OLS), with model coefficients shown at the top of the figure. The association between publication year and the amount of citation is statistically significant ($P < 0.0001$), with earlier-published cell lines tending to have more citations

Figure S2. hPSC across Jurisdictions Identified in Cellosaurus. The X-axis represents 28 jurisdictions identified from the Cellosaurus database, and the Y-axis shows the number of hPSC lines associated with each jurisdiction

Figure S3. Data sources for Australian hPSCs documented in Cellosaurus. Venn diagram shows the three key data sources—hPSCreg, NIHhESC, and SKIP—used by Cellosaurus to capture hPSC lines originating from Australia.

Table S1. Detailed Framework for FAIR Evaluation of hPSC Data Infrastructures. The left column detailed 15 guiding FAIR principles from GO FAIR (2025) and Wilkinson et al. (2016). The corresponding “Assessment Criteria” column on the right presents our translated evaluation framework for stem cell data infrastructures. Principles shown shaded in grey represent those that are challenging to assess or not directly applicable to stem cell infrastructures

Table S2. Integration of ICSCB and Cellosaurus Data Fields Illustrated by H9 Records. (See in the excel file)

Table S3. Completeness of Data Fields in Cellosaurus. This table presents 15 main and 13 subfields from Cellosaurus that are relevant to stem cell data, organized by ISSCR information categories. Completeness rates are calculated for each field and color-coded as follows: green (>90%), yellow (20–90%), and red (<20%).

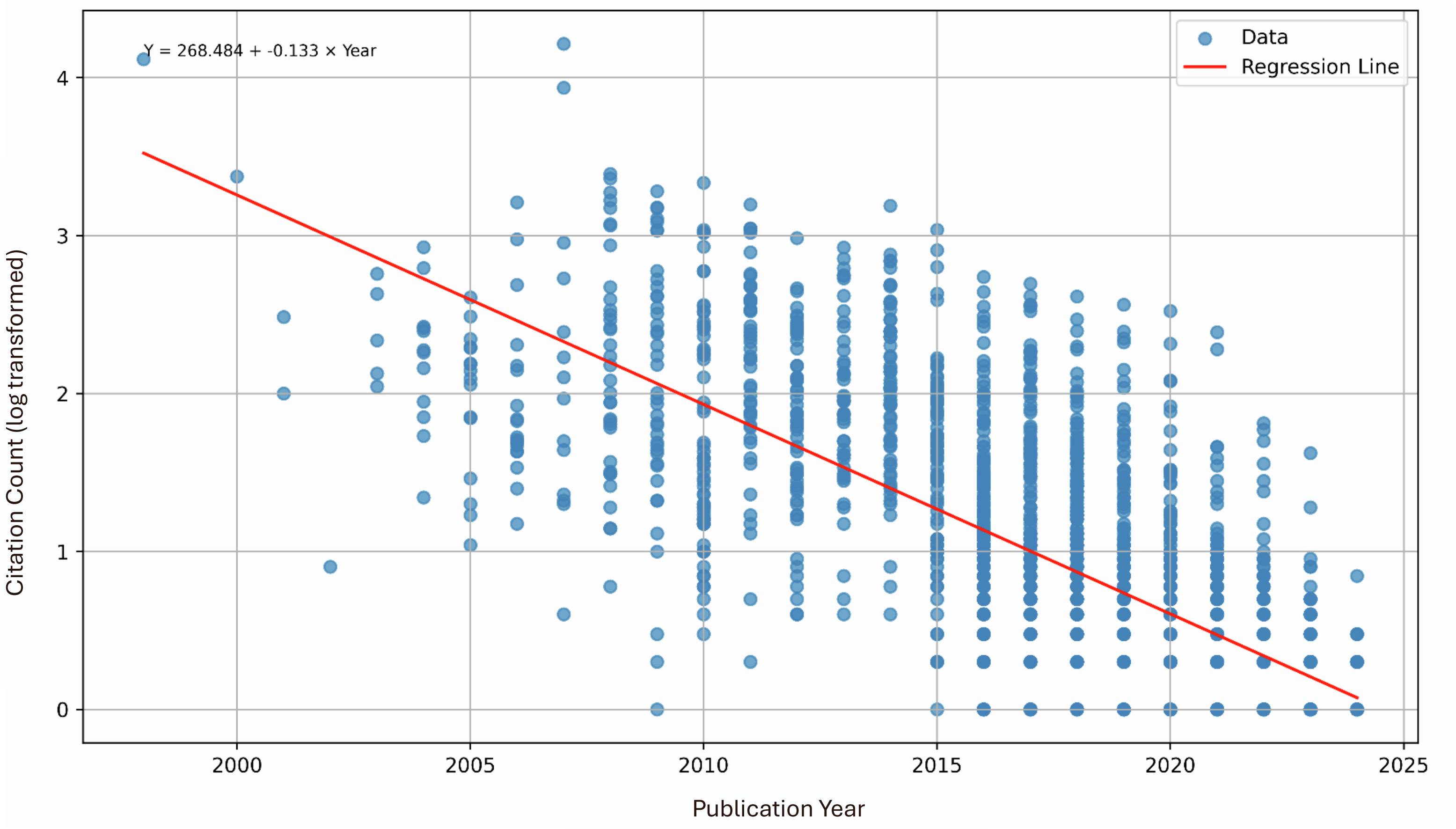

Figure S1. Effect of Time (Publication Year) on Citation Count of Cell Lines. Citations of published cell lines are shown in a dot plot, with the Y-axis representing publication year and the X-axis representing citation count. Citation counts were log-transformed ($\log_{10} + 1$) to ensure all values are positive. A linear regression line (red) was fitted using Ordinary Least Squares (OLS), with model coefficients shown at the top of the figure. The association between publication year and the amount of citation is statistically significant ($P < 0.0001$), with earlier-published cell lines tending to have more citations.

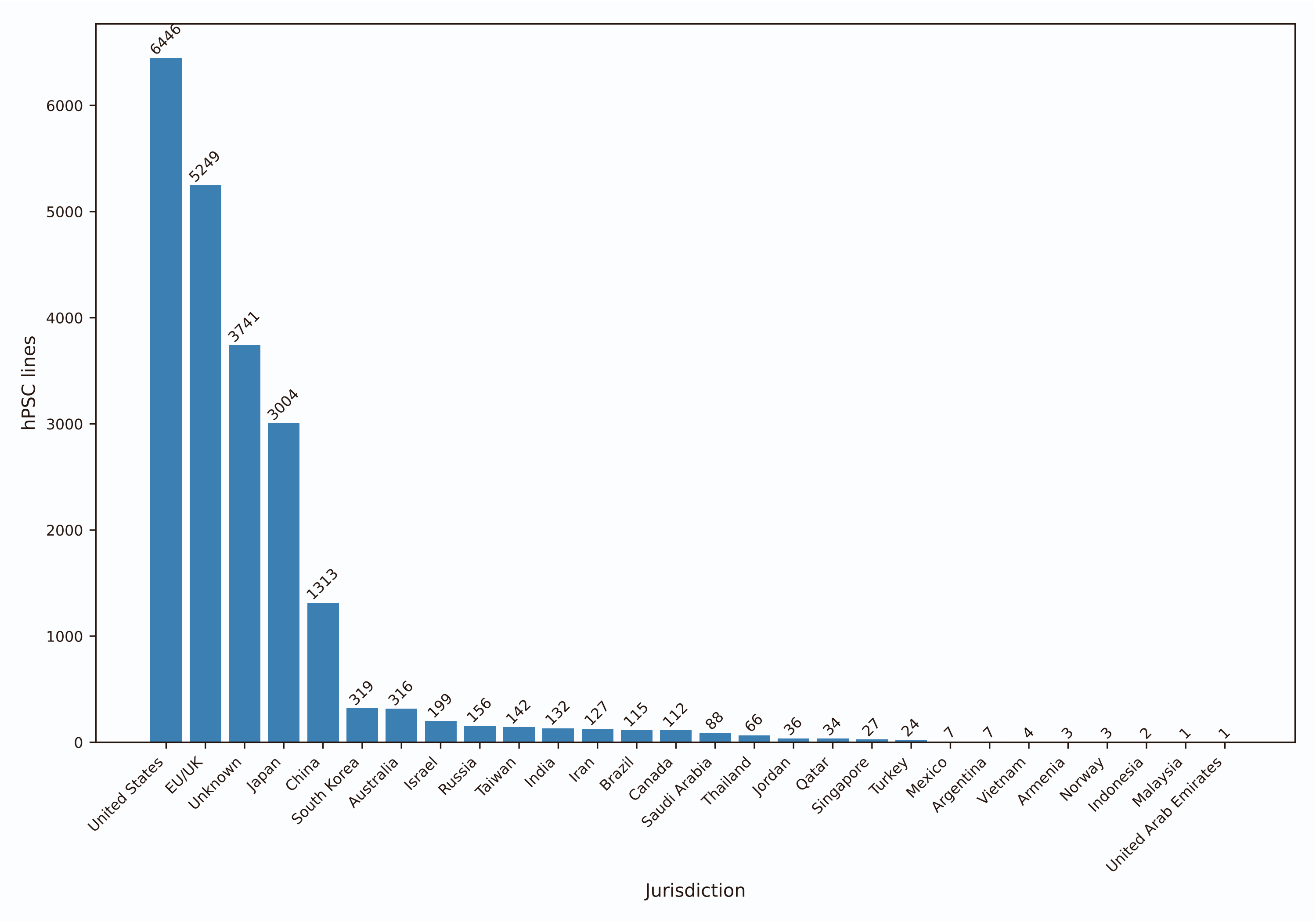

Figure S2. hPSC across Jurisdictions Identified in Cellosaurus. The X-axis represents 28 jurisdictions identified from the Cellosaurus database, and the Y-axis shows the number of hPSC lines associated with each jurisdiction.

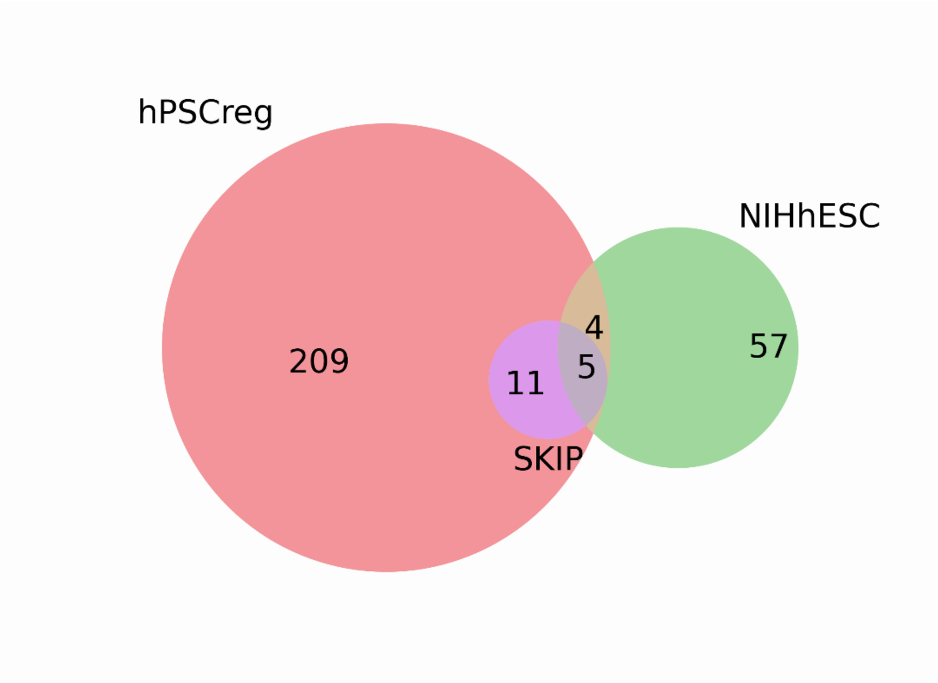

Figure S3. Data sources for Australian hPSCs documented in Cellosaurus. Venn diagram shows the three key data sources—hPSCreg, NIHhESC, and SKIP—used by Cellosaurus to capture hPSC lines originating from Australia.

Table S1. Detailed Framework for FAIR Evaluation of hPSC Data Infrastructures. The left column detailed 15 guiding FAIR principles from GO FAIR (2025) and Wilkinson et al. (2016). The corresponding "Assessment Criteria" column on the right presents our translated evaluation framework for stem cell data infrastructures. Principles shown shaded in grey represent those that are challenging to assess or not directly applicable to stem cell infrastructures.

<table>
<tr><th colspan="3">FAIR Guiding Principles (Go Fair, 2025; Wilkinson et al., 2016a)</th><th>Assessment Criteria</th></tr>
<tr><td rowspan="4">Findable (F)
The first step in (re)using data is to find them. Metadata and data should be easy to find for both humans and computers. Machine-readable metadata are essential for the automatic discovery of datasets and services, so this is an essential component of the FAIRification process.</td><td colspan="2">F1. (Meta)data are assigned a globally unique and persistent identifier.</td><td>Is a globally unique and persistent identifier assigned to the cell line metadata in this infrastructure?</td></tr>
<tr><td colspan="2">F2. Data are described with rich metadata (defined by R1 below).</td><td>(See R1)</td></tr>
<tr><td colspan="2">F3. Metadata clearly and explicitly includes the identifier of the data they describe.</td><td>Are identifiers assigned to the cell lines described in the metadata?
Does the metadata in this infrastructure cross-link to other (meta)data?</td></tr>
<tr><td colspan="2">F4. (Meta)data are registered or indexed in a searchable resource</td><td>Is metadata searchable within the infrastructure?</td></tr>
<tr><td rowspan="3">Accessible (A)
Once the user finds the required data, she/he/they need to know how they can be accessed, possibly including authentication and authorisation.</td><td rowspan="2">A1. (Meta)data are retrievable by their identifier using a standardised communications protocol.</td><td>A1.1 The protocol is open, free, and universally implementable.</td><td rowspan="2">Is metadata accessible from the infrastructure?
Can the metadata be downloaded in a machine-readable format?</td></tr>
<tr><td>A1.2 The protocol allows for an authentication and authorisation procedure, where necessary.</td></tr>
<tr><td colspan="2">A2. Metadata is accessible, even when the data are no longer available</td><td>Is the metadata still accessible when the infrastructure is retired?</td></tr>
<tr><td rowspan="3">Interoperable (I)
The data usually needs to be integrated with other data. In addition, the data needs to interoperate with applications or workflows for analysis, storage, and processing.</td><td colspan="2">I1. (Meta)data use a formal, accessible, shared, and broadly applicable language for knowledge representation.</td><td>How does the use of PID affect metadata integration and comparison across other platforms?
How does data structure affect integration and comparison across other platforms?</td></tr>
<tr><td colspan="2">I2. (Meta)data use vocabularies that follow FAIR principles.</td><td>How do data standards impact data integration and comparison across other platforms?</td></tr>
<tr><td colspan="2">I3. (Meta)data include qualified references to other (meta)data.</td><td>-</td></tr>
<tr><td rowspan="3">Reusable (R)
The ultimate goal of FAIR is to optimise the reuse of data. To achieve this, metadata and data should be well-described so that they can be replicated and/or combined in different settings.</td><td rowspan="3">R1. (Meta)data are richly described with a plurality of accurate and relevant attributes.</td><td>R1.1. (Meta)data are released with a clear and accessible data usage license</td><td>-</td></tr>
<tr><td>R1.2. (Meta)data are associated with detailed provenance.</td><td>Does the metadata include information about the provenance of cell lines?</td></tr>
<tr><td>R1.3. (Meta)data meet domain-relevant community standards.</td><td>Does the metadata contain a lot of missing values?
Does the metadata in this infrastructure comply with ISSCR guidelines?</td></tr>
</table>

Table S2. Integration of ICSCB and Cellosaurus Data Fields Illustrated by H9 Records. (See in the excel file)

Table S3. Completeness of Data Fields in Cellosaurus. This table presents 15 main and 13 subfields from Cellosaurus that are relevant to stem cell data, organized by ISSCR information categories. Completeness rates are calculated for each field and color-coded as follows: green (>90%), yellow (20–90%), and red (<20%).

ISSCR Category	Field Type	Field Name	Explanation	Completeness Rate
Basic Characteristics	Main	AC	Cellosaurus accession number	100%
	Main	ID	Cell line original ID	100%
	Main	DT	Date of creation, last updated, version	100%
	Main	DR	Cross-references to external databases	100%
	Main	CA	Type of stem cell (iPSC/ESC)	100%
	Main	SY	List of synonyms	71%
	Subfield	From	Institution of origin	65%
	Main	RX	References IDs for publication or patent	54%
	Main	OI	Derived from the same individual	31%
	Main	HI	Parental or descendant cell lines	25%
	Subfield	Discontinued	Not available in a bank	14%
	Subfield	Part of	Part of a bank, study or consortium	10%
	Main	ST	Short tandem repeat (STR) profile	6%
	Subfield	Registration	Registered or banked infrastructures	4%
	Subfield	Group	Clinical-grade or patented status	0%
	Subfield	Problematic cell line	Misidentified, contaminated, or from a retracted paper	0%
Stem Cell-Based Model Systems	Main	SX	Donor's sex	97%
	Main	AG	Donor's age at sampling	93%
	Subfield	Derived from site	Tissue sites	91%
	Subfield	Cell type	Original tissue type	59%
	Main	DI	Associated diseases	48%
	Subfield	Population	Ethnicity	45%
Genomic Characterization	Subfield	Sequence variation	Mutation details	23%
	Subfield	Characteristics	Genetic modificatiosn	12%
	Subfield	Omics	Types of omics analysis performed	12%
	Subfield	Knockout cell	Gene knockout information	2%
	Subfield	HLA typing	HLA typing data	2%
	Subfield	Karyotypic information	Karyotypes	0%
