# Supplementary material for: How FAIR is metadata for human pluripotent stem cells?
Source: Stem Cell Reports. 2025 Sep 25;20(10):102644. doi: 10.1016/j.stemcr.2025.102644 (PMC12790715; doi:10.1016/j.stemcr.2025.102644)
Supplement: Document S2. Article plus supplemental information [file mmc3.pdf]

Stem Cell Reports
**Article**
OPEN ACCESS

# How FAIR is metadata for human pluripotent stem cells?

Mengqi Hu,[1] Rachel A. Ankeny,[2,3] Dan Santos,[4] and Christine A. Wells[1,5,*]
[1]Stem Cell Systems, The Department of Anatomy and Physiology, Faculty of Medicine, Dentistry and Health Sciences, The University of Melbourne, Parkville, VIC 3010, Australia
[2]School of Humanities, University of Adelaide, Napier, Adelaide, SA 5005, Australia
[3]Wageningen University, Hollandseweg 1, 6706 KN Wageningen, the Netherlands
[4]Australian National Centre for the Public Awareness of Science, Australian National University, Acton, ACT 2601, Australia
[5]Lead contact
*Correspondence: wells.c@unimelb.edu.au
https://doi.org/10.1016/j.stemcr.2025.102644

## SUMMARY

The rapid and global expansion of stem cell research over the last two decades necessitates coordinated and effective management of information describing stem cell lines and accompanying data resources. Here, we evaluate the maturity of the field by applying FAIR data principles—findable, accessible, interoperable, and reusable—to assess the quality of information describing human pluripotent stem cells (hPSCs) in dedicated data infrastructure. We identified a lack of coordination across different jurisdictions that prevents effective information sharing, such as the absence of persistent digital identifiers, inconsistent data standards, and restrictive sharing policies. Using Australia, the United States, Japan, and Europe as case studies, we underscore the need for national infrastructure to support comprehensive cell line cataloging. This is the first systematic evaluation of FAIR principles in the field and indicates that improving metadata standardization and cross-platform coordination will enhance data reuse and strengthen the value of local resources.

## INTRODUCTION

The scale and complexity of scientific data have grown rapidly in recent years (Baysoy et al., 2023). In stem cell science, the advent of induced pluripotent stem cell (iPSC) technology, facilitated by accessible derivation from tissues such as skin (Takahashi et al., 2007; Yu et al., 2007) and blood (Staerk et al., 2010) has led to a surge in stem cell lines and associated data. Conservative estimates suggest that over 20,000 human pluripotent stem cells (hPSCs), including iPSC and embryonic stem cells (ESCs), have been generated worldwide (Bairoch, 2018; Wells et al., 2024). This rapid expansion highlights the urgent need for effective data management to ensure provenance and reproducibility.

The FAIR data principles—findable, accessible, interoperable, and reusable—were first introduced by the genomics community as a response to growing challenges in data management, with 15 guidelines that emphasize comprehensive metadata documentation (Wilkinson et al., 2016). Since then, initiatives such as GO FAIR (GO, 2016), the European Open Science Cloud (European Commission, 2015), and the Australian Research Data Commons (Australian Research Data Commons, 2018), along with major funding bodies including the European Commission (EC) and the National Institutes of Health (NIH), have promoted FAIR compliance (reviewed in Wang and Savard, 2023). Relevance to the stem cell community was emphasized by the incorporation of FAIR principles into the 2023 International Society for Stem Cell Research (ISSCR) guidelines (Ludwig et al., 2023).

While the FAIR principles offer a broad framework, they do not prescribe specific implementation strategies, making their application context dependent (Jacobsen et al., 2020). In stem cell research, this requires attention to the unique characteristics of hPSCs, which exhibit diverse biological phenotypes. Capturing this complexity depends on rich metadata—such as donor attributes, derivation methods, and culture conditions. Although no universally enforced standards exist for stem cell metadata reporting, the ISSCR's 2023 guidelines offer a widely accepted, practical framework. They define four key metadata categories: basic characteristics (e.g., identifiers, source tissue, and derivation), pluripotency and the undifferentiated state (evidence of pluripotency), genomic characterization (genetic variations), and stem cell-based model systems (including differentiation potential and donor metadata) (Ludwig et al., 2023). Moreover, registration within dedicated infrastructures is considered a key step in FAIRifying stem cell data, as it enables linking information about a line's origin with subsequent data generated using that line (Ludwig et al., 2023; Seltmann et al., 2016). Biobanks and data registries have been developed to capture, store, and disseminate such metadata, and many of these aim to be FAIR compliant. For example, the human Pluripotent Stem Cell Registry (hPSCreg, Seltmann et al., 2016) has implemented "persistent identifiers" (PIDs), a core FAIR principle, to provide unique and stable references to digital objects. hPSCreg uses a nomenclature specifically designed for hPSCs (Kurtz et al., 2018). Cellosaurus cell


Published by Elsevier Inc. on behalf of International Society for Stem Cell Research.
This is an open access article under the CC BY license (http://creativecommons.org/licenses/by/4.0/).

**Table 1. Framework for FAIR evaluation of hPSC data infrastructures**

| Category | Focus | FAIR principles |
|---|---|---|
| Record access | ● is cell line record still accessible if the database is no longer maintained?<br>● is cell line record accessible and downloadable? | accessible (A1, A2) |
| Identifier | ● are PIDs assigned?<br>● is cell line record linked to external databases?<br>● is cell line record searchable? | findable (F1, F3, F4) |
| Data interoperability | ● are metadata using shared vocabularies, PIDs, and standards that enable integration across platforms? | interoperable (I1, I2) |
| Metadata quality | ● are metadata rich, complete, and aligned with ISSCR standards? | findable (F2) & reusable (R1.2, R1.3) |

line database (Bairoch, 2018) supports a broader range of cell types and is tasked by the NIH-funded Research Resource Identification (RRID) project to assign PIDs to cell lines in the public domain (Bandrowski et al., 2015). While Cellosaurus lacks many stem cell-specific fields, it does draw heavily from hPSCreg and other databases to populate metadata information about stem cell lines. Despite these and other stem cell-aligned databases, there has been no formal evaluation of how key metadata fields suggested by the ISSCR standards align with information collated by each resource, nor how these map onto the 15 FAIR principles for data governance.

We argue that reviewing the alignment of digital infrastructures across jurisdictions is both timely and important—especially as the development of regional data systems is increasingly shaped by how funding and regulatory bodies respond to the broader open science movement (Fecher and Friesike, 2014). For example, countries in the European Union, the United Kingdom (UK), the United States (US), and Japan have adopted both stem cell data infrastructures and FAIR principles in a relatively coordinated manner. Europe, where FAIR originated, has integrated the principles into research policy, requiring data management plans for all EC-funded projects and supporting stem cell registration through hPSCreg (European Commission, 2016). The US has similar FAIR practices through NIH policies, including the 2023 Data Management and Sharing Policy, which emphasizes the use of PIDs and standardized indexing tools (National Institute of Health, 2023). Japan, a global leader in hPSC research, has focused on the preservation of physical biomaterials through initiatives such as the National BioResource Project (NBRP), with RIKEN Biobank as a key node for hPSCs (MEXT, 2002). In addition, countries such as Iran (Royan institute, 2020), Brazil (Martins de Oliveira et al., 2023), China (Wang et al., 2021), and South Korea (Kim et al., 2021) have established stem cell biobanks or registries to support biomedical research and data sharing, though with varying degrees of FAIR alignment. In contrast, Australia lacks a dedicated stem cell infrastructure, and interviews with researchers reveal limited awareness of international registries and engagement in registration practices (Hu et al., 2024). However, efforts are underway to promote FAIR data principles nationally, with a particular focus on the use of PIDs (Australian Research Data Commons, 2024). These efforts are further reinforced by stem cell-related journals such as *Nature*, *Cell*, and *Science*, which require the adoption of RRIDs for resources, including cell lines (Bandrowski et al., 2015).

This study evaluates how well digital infrastructures supporting hPSC research align with FAIR data principles, as a reflection of the field's progress toward open science practices. By comparing infrastructures across Australia, Europe, the US, and Japan, we highlight regional differences in data governance and identify key gaps and opportunities for improvement. Based on these findings, we propose recommendations for researchers, journals, funders, and institutions to strengthen FAIR data governance and enhance the discoverability, consistency, and reuse of stem cell data globally.

## RESULTS

### Challenges in achieving FAIR compliance for hPSC metadata

We interpreted the 15 FAIR guiding principles in the context of hPSCs and developed a tailored evaluation framework to assess metadata documentation across stem cell data infrastructures worldwide (Table 1, details in Table S1). In total, 28 digital resources were identified (Table 2), including 20 biobanks, 6 data registries, and 2 integrated databases (Cellosaurus and ICSCB). We obtained information from digital resources using the website uniform resource locators (URLs) and manually searched for metadata files, downloadable objects, or Application Programming Interface (API). This process informed the subsequent structure of our analysis.

**Table 2. Stem cell digital infrastructures reviewed in this study**

| Jurisdiction | Type | Name | URL | Cellosaurus | ICSCB |
|---|---|---|---|---|---|
| Europe | registry | Human Pluripotent Stem Cell Registry (hPSCreg) | https://hpscreg.eu/ | ✓ | ✓ |
| | bank | European Bank for Induced pluripotent Stem Cells (EBiSC) | https://cells.ebisc.org/ | ✓ | – |
| | | Barcelona Stem Cell Bank (BLCB; Spain) | https://idibell.cat/en/services/scientific-and-technical-services/stem-cell-bank/ | – | – |
| | | Human Induced Pluripotent Stem Cells Initiative (HipSci; UK) | https://www.hipsci.org/ | ✓ | – |
| | | UK Stem Cell Bank (UK) | https://www.nibsc.org/ukstemcellbank | – | – |
| | | European Collection of Cell Cultures (ECACC) | https://www.culturecollections.org.uk/about-us/ecacc/ | ✓ | – |
| | database | Cellosaurus | https://www.cellosaurus.org/ | – | – |
| United States | registry | NIH Human Embryonic Stem Cell Registry (NIHhEC) | https://grants.nih.gov/stem_cells/registry/current.htm | ✓ | – |
| | | NINDS Human Cell and Data Repository (NHCDR) | https://stemcells.nindsgenetics.org/ | ✓ | – |
| | | Eagle-i (Retired) | https://open.catalyst.harvard.edu/products/eagle-i/ | – | ✓ |
| | | International Stem Cell Registry (ISCR; Retired) | https://www.umassmed.edu/ | – | – |
| | bank | CIRM-FujiFilm Cellular Dynamics, Inc (FCDI) | https://www.fujifilmcdi.com/search-cirm/ | ✓ | ✓ |
| | | Harvard Stem Cell Institute (HSCI) | https://hsci.harvard.edu/ | – | – |
| | | Coriell Institute Biorepositories (Coriell) | https://catalog.coriell.org | ✓ | – |
| | | Parkinson’s Progression Markers Initiative (PPMI) | https://www.ppmi-info.org/access-data-specimens/request-cell-lines | – | – |
| | | Cedars-Sinai | https://csbiomfg.com/cellcollection/#ipsclines | – | – |
| | | New York Stem Cell Foundation (NYSCF) | https://nyscf.org/research-institute/repository-stem-cell-search/ | – | – |
| | | Allan Institute for Cell Science (AICS) | https://www.allencell.org/cell-catalog.html | – | – |
| | | WiCell Research Institute (WiCell) | https://www.wicell.org/ | ✓ | – |
| | | American Type Culture Collection (ATCC) | https://www.atcc.org/ | ✓ | – |
| Japan | registry | Stem cell Knowledge and Information Portal (SKIP; Retired) | https://saiseiiryo.jp/skip_archive/ | ✓ | ✓ |
| | bank | RIKEN BioResource Research Center Cell Bank (RCB) | https://cell.brc.riken.jp/en/rcb | ✓ | ✓ |
| | | Japanese Collection of Research Bioresources Cell Bank (JCRB) | https://cellbank.nibiohn.go.jp/english/ | ✓ | – |
| | database | Integrated Collection of Stem Cell Bank (ICSCB) data | https://icscb.stemcellinformatics.org/ | – | – |
| China | bank | National Stem Cell Resource Center (CSCR) | http://www.nscrc.cn/ | – | – |

(*Continued on next page*)

**Table 2. *Continued***

| Jurisdiction | Type | Name | URL | Cellosaurus | ICSCB |
|---|---|---|---|---|---|
| Korea | bank | National Stem Cell Bank and Registry of Korea | https://www.nih.go.kr/ncsr/nscb/en/kscr/index.do | – | – |
| Taiwan | bank | Taiwan Bioresource Collection and Research Center (BCRC) | https://catalog.bcrc.firdi.org.tw/ | ✓ | – |
| Iran | bank | Royan Stem Cell Bank (RSCB; Retired) | https://web.archive.org/web/20201001144644/http://www.royaninstitute.org/cmsen/index.php?option=com_content&task=view&id=205&Itemid=40 | ✓ | – |

Ticks in the "Cellosaurus Capture" and "ICSCB Capture" columns indicate that the infrastructures' metadata are included in Cellosaurus and ICSCB, respectively.

## The lack of uniform data-sharing standards or controls across databases is not aligned with FAIR accessibility principles

According to the accessibility (A) principles of FAIRness, metadata need to be retrievable by their identifier using an open, free, and universally implementable protocol (A1), and they should remain accessible even when the data are no longer available (A2).

Our analysis of the 28 identified infrastructures revealed several challenges in meeting these principles. Four infrastructures—Eagle-i, the International Stem Cell Registry (ISCR), the Stem Cell Knowledge and Information Portal (SKIP), and the Royan Stem Cell Bank (RSCB)—are no longer active, and their websites were not able to be accessed at the time of this study (Table 2). The Californian Institute of Regenerative Medicine stem cell collection housed within the Fujifilm Cellular Dynamics biobank (CIRM-FCDI) has also ceased operations recently, although its website remains accessible (Canet-Avilés, 2025). The integrated resources Cellosaurus and ICSCB separately captured metadata from Eagle-i and RSCB, and both included SKIP. However, ISCR's metadata do not appear accessible through any resource, presenting a challenge for A2 compliance.

In addition to these inactive infrastructures, we found that six biobanks, including the Barcelona Stem Cell Bank (BLCB), UK Stem Cell Bank (UKSCB), Harvard Stem Cell Institute, National Stem Cell Bank and Registry of Korea, Parkinson's Progression Markers Initiative (PPMI), and National Stem Cell Resource Center (CSCR), lack an online catalog that gave FAIR-aligned metadata for individual cell lines. Instead, these sites provide only general information or summary statistics for their entire collection, requiring direct contact with the infrastructure staff for specific information, creating a barrier to A1 compliance.

Inconsistent metadata-sharing controls across infrastructures present a challenge to FAIR accessibility. Some platforms impose restricted access to metadata (such as those listed earlier), while others allow access to individual cell line records but limit bulk downloads. For example, most infrastructures, including the European Bank for Induced Pluripotent Stem Cells (EBiSCs), Human Induced Pluripotent Stem Cells Initiative (HipSci), and the NIH Human Embryonic Stem Cell Registry (NIHhEC), do not support batch data downloads. In contrast, hPSCreg enables metadata sharing via an API, and both Cellosaurus and ICSCB allow full database downloads in machine-readable formats. As Cellosaurus and ICSCB collate information from various sources, we also examined whether information from individual repositories was captured by these database aggregators (Table 2). These two approaches—examining data records within individual databases or evaluating them after aggregation in centralized databases—served as the basis for assessing FAIR alignment.

## Limited adoption of persistent digital identifiers impacts cell line "findability"

Findability in the FAIR guidelines specifies that (meta)data should be assigned a PID (F1). Eight infrastructures identified in the previous section were excluded from further assessment due to data inaccessibility, which made it impossible to determine whether or how identifiers were used.

Among the remaining 20 infrastructures, only five use PIDs. Four—EBSiSC, HipSci, European Collection of Cell Cultures (ECACC), and WiCell—incorporate the hPSCreg nomenclature, while Cellosaurus uses RRID. The other 15 infrastructures rely solely on platform-specific local identifiers and do not reference external (meta)data. The American Type Culture Collection (ATCC) is a partial exception, as it provides links to relevant publications. Integrating metadata from platforms that lack PIDs requires substantial manual effort and a bit of curatorial judgment to verify the identity of cell lines appearing

across multiple databases. As Cellosaurus and the Integrated Collection of Stem Cell Bank (ICSCB) data are aggregators that preserve local identifiers, we used them to assess how well cell line information can be integrated across distinct data resources.

ICSCB retains local identifiers from contributing platforms but does not appear to check for duplicate entries (Chen et al., 2021). After removing nine clear duplicates, we compared 16,462 ICSCB records with 21,674 from Cellosaurus and found that 65% ($n$ = 10,781) were already present in Cellosaurus. Additionally, 400 ICSCB entries matched multiple Cellosaurus records (349 ICSCB entries matched two Cellosaurus records; 50 matched three, and one matched four). We suspect that these overlapping records were a consequence of multiple rounds of data aggregation. This issue is further complicated by the fact that most of the remaining ICSCB entries, which appeared unique, originated from Eagle-i ($n$ = 2,400), RIKEN Biobank ($n$ = 1,973), hPSCreg ($n$ = 698), and SKIP ($n$ = 610)—all of which are also data sources aggregated by Cellosaurus. Therefore, without PIDs, it is difficult to determine whether entries across platforms represent distinct cell lines or duplicates, highlighting the challenges of cross-database integration.

Although all cell lines in these infrastructures meet the FAIR F4 guideline by being searchable within their own platforms, they are not universally discoverable due to the absence of PIDs. This is concerning, as the lack of coordinated digital identifiers across databases increases the risk of non-unique IDs being assigned to different cell lines, leading to confusion about their provenance or identity.

## Factors preventing interoperability across stem cell databases

The principles of interoperability (I) primarily assess whether data can be integrated with other datasets and how easy or difficult this process is. As previously noted, the lack of standardized PIDs makes it difficult to determine whether records refer to the same entity. Beyond identifiers, assessing interoperability also involves examining the specifics of the information infrastructures provide.

We used the well-known hESC line H9 (RRID: CVCL_9773) as a case study to compare metadata in Cellosaurus and ICSCB. Most ICSCB's fields (13/14), including donor age, ethnicity, sex, provider, and associated publications, were also captured in Cellosaurus (Table S2). However, field names often differed. For instance, Cellosaurus uses abbreviated labels (e.g., donor age is recorded as "AG"), while ICSCB uses more descriptive terms such as "age_of_donor." Some information is split across multiple fields in one platform but combined in the other; for example, Cellosaurus uses a single "DR" field for both the source infrastructure and local ID, whereas ICSCB separates them into "_source" and "_cellid." Field values also varied. For example, Cellosaurus records tissue origin as "blastocyst," while ICSCB uses the broader term "Fresh Embryo." Differences in data structure further complicated integration; for example, Cellosaurus records tissue of origin as a list, while ICSCB captures it as a single string. These inconsistencies in field design, vocabulary, and data structure introduce unnecessary computational barriers and risk propagating inaccurate or conflicting information.

## Reusability is aligned with database implementation of community standards

Proper metadata documentation is essential to enable replication and integration in different settings (R). Beyond provenance (R1.2), we argue that detailed metadata aligned with domain-relevant community standards (R1.3) are also critical for ensuring reusability. To evaluate this, we focused on Cellosaurus, as it includes information from hPSCreg and ICSCB and has a fully downloaded database. We compared Cellosaurus metadata fields against ISSCR standards and examined field completeness rates for hPSC lines (Table 3).

Our analysis showed relatively good coverage of "Basic Characteristics" and "Stem Cell-Based Model Systems" but limited documentation for "Genomic Characterization" and none for "Pluripotency and the Undifferentiated State"—the latter captured by registries like hPSCreg. While we recognize that not all fields are expected to be completed for every cell line (e.g., the associated disease does not apply to healthy donors), we observed that many key fields lack sufficient completeness. Important metadata, such as cell line origin ("From"); associated infrastructures ("Registration"); and donor details like sex ("SX"), age ("AG"), tissue source ("Cell type"), and disease information ("DI"), were frequently missing. Other valuable fields, including short tandem repeat profiles and self-reported ethnicity, also show low documentation rates.

One important question is how FAIR-aligned metadata practices might influence the reuse of cell line information. Our evaluation indicates that, although stem cell records are captured in FAIR data infrastructure such as Cellosaurus, the lack of FAIR alignment with source databases feeding into Cellosaurus can impact the clarity, interoperability, and overall usefulness of these records to researchers. Addressing these gaps calls for more than technical fixes. It requires the community to define how "open" regional infrastructure should be in sharing basic cell line information, agreeing to common formats for registration of cell lines, promoting the adoption of PIDs, and agreeing on community standards for data harmonization and

**Table 3. Completeness of the data fields in Cellosaurus**

| ISSCR Category | Completeness Rate | Field Summary |
|---|---|---|
| Basic Characteristics | 100% | Accession ID, Registration date, Cross-references |
| | 25-71% | Synonyms, Institution, References, Parental/descendant lines |
| | 0-4% | STR profile, Biobank, Patent/clinical-grade info, Problematic lines |
| Stem Cell-Based Model Systems | 91-97% | Sex, Age, Tissue source |
| | 45-59% | Cell type, Disease, Population |
| Genomic Characterization | 23% | Mutation details |
| | 0-12% | Omics, Karyotyping, HLA typing, Knockouts, Genetic modification |
| Pluripotency and the Undifferentiated State | - | None reported |

Data fields are color-coded according to completeness: green indicates >90% completeness, yellow 20%–90%, and red <20%.

integration. Ultimately, it requires a collective commitment of cell line generators and data wranglers to align data practices across infrastructures.

## Most hPSC lines are not shared widely

The ultimate goal of FAIR principles is to optimize data reuse (Wilkinson et al., 2016). While our earlier analysis focused on conceptual alignment, we complemented it by examining the practical reuse of stem cell lines through publications linked to cell lines in Cellosaurus. Since experimental research depends on the physical reuse of cell lines, tracking their use provides a direct measure of reusability. We quantified this using traditional impact metrics—publication and citation counts.

We used the number of publications associated with each hPSC line as a proxy for its reuse across studies. Among 21,674 hPSCs in Cellosaurus, 54% ($n$ = 11,675) were linked to PubMed-indexed publications (Figure 1A). The top five publishing journals (Figure 1B) were *Stem Cell Research* (31%), *Cell Reports* (19%), *Nature* (9%), *Cell Stem Cell* (6%), and *Stem Cell Reports* (6%). Publication counts were highly right skewed (skewness = 14.5; kurtosis = 393), with a median of 1 and a mean of 1.16 per line (Figure 1C). Notably, 89% ($n$ = 10,430) of lines were associated with only one publication and 99% with three or fewer. A small subset of highly published lines ($\geq$10 publications) accounted for a disproportionate share of reuse. These included some of the earliest described ESC and iPSC lines, such as *H1* Cellosaurus identifier *(CVCL_9771)*, *H9 (CVCL_9773)*, *201B7 (CVCL_A324)*, *H7 (CVCL_9772)*, *585A1 (CVCL_DQ06)*, *KhES-3 (CVCL_B233)*, and *H14 (CVCL_9775)*.

It is possible that publication alone is sufficient for "Findabilty" of stem cell lines, particularly given journal formats such as that in *Stem Cell Research* (the top indexed journal in Cellosaurus) that are designed to report on new lines. To assess impact of published stem cell studies, we analyzed citation counts of publications associated with each cell line. Among the 11,675 hPSCs associated with a publication, 13% ($n$ = 1,544) had no recorded citations in the Scopus database. This included 108 lines for which publication metadata could not be retrieved via their PubMed ID. The average number of citations per hPSC was 169; however, the distribution was highly skewed (median = 20, skewness = 16.1; kurtosis = 290), with only a small proportion of cell lines being very highly cited (Figure 1C). Specifically, 28 lines (0.2%) were cited >10,000 times, and 319 lines (2.7%) were cited between 1,000 and 10,000 times. Additionally, 24% ($n$ = 2,753) had 1–10 citations, 38% ($n$ = 4,404) had 10–100, and 23% ($n$ = 2,627) were cited between 100–1,000 times. The most highly cited lines included five of the previously mentioned lines *201B7 (CVCL_A324)*, *H1 (CVCL_9771)*, *H9 (CVCL_9773)*, *H7 (CVCL_9772)*, and *H14 (CVCL_9775)*, along with *201B6 (CVCL_A065)* and *246G1 (CVCL_C243)*. As these are also among the first hPSCs made, we expect that citation count is heavily influenced by time. Indeed, publication year has a significant effect on citation count—papers published earlier (i.e., with a smaller year value) tend to receive more citations (Figure S1).

Given most published lines are not highly cited, we next assessed whether registration or cell line banking had any impact on cell line reuse. Here, we assume that a line associated with multiple publications is an indication of reuse across different studies (Figure 1D). Compared to the 61% registration or banking rate among reference lines that were only described in a single publication, we observed a higher likelihood of registration and banking with increased reuse: 63% for lines published in two studies, 83% for those in three or four studies, and

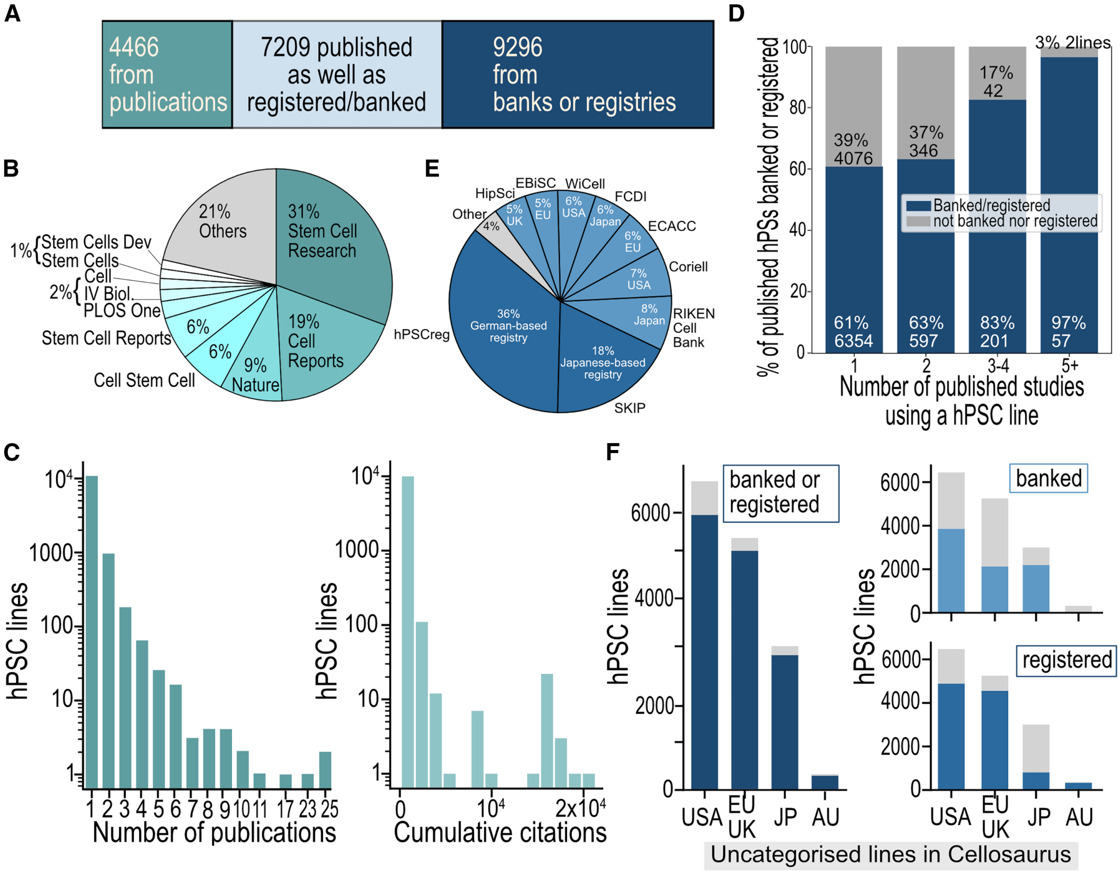


**Figure 1. Overview of stem cell infrastructures and publication for Cellosaurus hPSCs**

(A) Rectangular Venn diagram showing the cross-referencing of Cellosaurus hPSCs by infrastructure and publication; (B) top ten published journals for Cellosaurus hPSCs; (C) publication count and citation count for published hPSC lines.

(D) Cell line reuse across multiple publications. Lines used across multiple studies (3–4 or 5+) were more likely to be banked or registered than lines published in a single study.

(E) Breakdown of stem cell banks and registries with cross-referenced infrastructures for Cellosaurus hPSCs; (F) overview of banking and registration by region: United States (US), Europe including the United Kingdom (EU/UK), Japan (JP), and Australia (AU).

97% for lines in more than five publications. We also found that lines that had been reused frequently (i.e., three or four studies) were significantly more likely to be registered or banked compared to reference lines with a log odds ratio of 3.07 ($p = 4.04 \times 10^{-13}$). This association was even stronger for cell lines heavily reused (i.e., five or more studies) with a log odds ratio of 18.28 ($p = 3.3 \times 10^{-10}$). This may indicate that these lines are more readily shared or, alternatively, that the lines have taken on high community importance and so are more likely to be banked or registered.

## While global infrastructures centralize data, national planforms ensure broader local coverage

Cellosaurus collated information from three registries and 12 banks, collectively covering 76% ($n$ = 16,505) of all hPSCs (Figure 1E). The three registries (hPSCreg, SKIP, and NHCDR) accounted for 56% of total hPSC entries, referencing 13,084 hPSCs. In contrast, the 12 banks contributed 44%, referencing 8,404 hPSCs. Notably, hPSCreg alone represented 36% of all hPSC entries in Cellosaurus and 45% of those recorded in other registries and banks.

Cellosaurus drew information from stem cell infrastructures—most of which, based on our findings, are predominately based in the US, Europe, or Japan (Table 2)—as well as from researcher-submitted data and the published literature (Bairoch, 2018). We wondered how regional differences in hPSC generation, registration, and banking practice impacted the findability of hPSC in global databases like Cellosaurus. For example, we were interested in comparing well-resourced regions to Australia, which has an active stem cell research community but lacks national infrastructure in this space. The US ($n$ = 6,446), Europe

**Table 4. Cellosaurus hPSC in the US, Europe, and Japan: Percentage coverage by hPSCreg vs. national infrastructures**

| | Cellosaurus hPSC | | National infrastructure coverage | | | hPSCreg coverage | |
|---|---|---|---|---|---|---|---|
| Jurisdiction | Total | Unpublished | Registry/bank | Total | Unpublished | Total | Unpublished |
| US | 6,446 | 4,203 | Coriell | 62% | 72% | 44% | 48% |
| | | | FCDI | | | | |
| | | | ATCC | | | | |
| | | | WiCell | | | | |
| | | | NIHhESC | | | | |
| | | | NHCDR | | | | |
| EU/UK | 5,249 | 2,376 | ECACC | 93% | 97% | 85% | 93% |
| | | | EBiSC | | | | |
| | | | HipSci | | | | |
| | | | hPSCreg | | | | |
| Japan | 3,004 | 2,221 | SKIP | 84% | 95% | 12% | 6% |
| | | | RCB | | | | |
| | | | JCRB | | | | |

hPSCreg is categorized here as both an international resource and an EU/UK infrastructure due to its origins in the EU.

($n = 5,249$), and Japan ($n = 3,004$) emerged as the top three jurisdictions contributing hPSCs entries to Cellosaurus (Figure 1F). A full breakdown of the 28 jurisdictions is presented in Figure S2. Among the top three regions, Europe leads in registration rates (87%), followed by the US (75%) and Japan (27%), while banking is more common in Japan (73%) and the US (60%), compared to Europe (41%), as illustrated in Figure 1F. At the time that this study was conducted, just 316 hPSC lines from Australia were visible in Cellosaurus, making this the sixth-ranking country in terms of hPSC generation. Our previous research, where we interviewed Australian researchers, estimates that over 1,300 hPSCs have been made by Australian laboratories, but most of these are not registered (Hu et al., 2024). Cellosaurus collated Australian lines primarily from data drawn from hPSCreg, SKIP, and the NIH hESC Registry (Figure S3).

As an international registry, hPSCreg may be preferred by some researchers over local resources for registering hPSC lines. To test this, we compared the percentage of hPSCs captured by jurisdictional infrastructures in the US, Europe, and Japan with that of hPSCreg (Table 4). We found that national infrastructures consistently showed broader coverage. For example, in Japan, national resources captured 84% of lines compared to 12% for hPSCreg. Similar patterns were seen in the US (62% vs. 44%) and Europe (93% vs. 85%), though with smaller gaps. We further examined unpublished hPSCs and observed the same trend: national registries and banks captured a larger share than hPSCreg across all three regions (Table 4). These data collectively indicate the importance of local investment in stem cell banks and registries but equally highlight the opportunities for improved data exchange between these resources to maximize the findability of information about a stem cell line. These data collectively underscore that gaps in registration or banking led to poor international visibility in FAIR science networks. Investment in regional/national infrastructure is likely accompanied by training and uptake in FAIR data practices.

## Stem cell infrastructure development in the US, Europe, Japan, and Australia

The history of stem cell research in the US, Europe, and Japan shows continual support and development of digital infrastructures in these regions. In total, two major waves of registry or banking infrastructure can be observed globally (Figure 2), aligning with the emergence of hESCs in 1998 and hiPSCs in 2007.

The first wave focused on the establishment of stem cell banks. Examples include WiCell, the hESC node in RIKEN Biobank, the UKSCB, and the Barcelona Stem Cell Bank. While Australia was considered a national bank during early legislation and policy discussions (Australian Government, 2005), it adopted a license-based system for regulating hESC research instead, without developing a

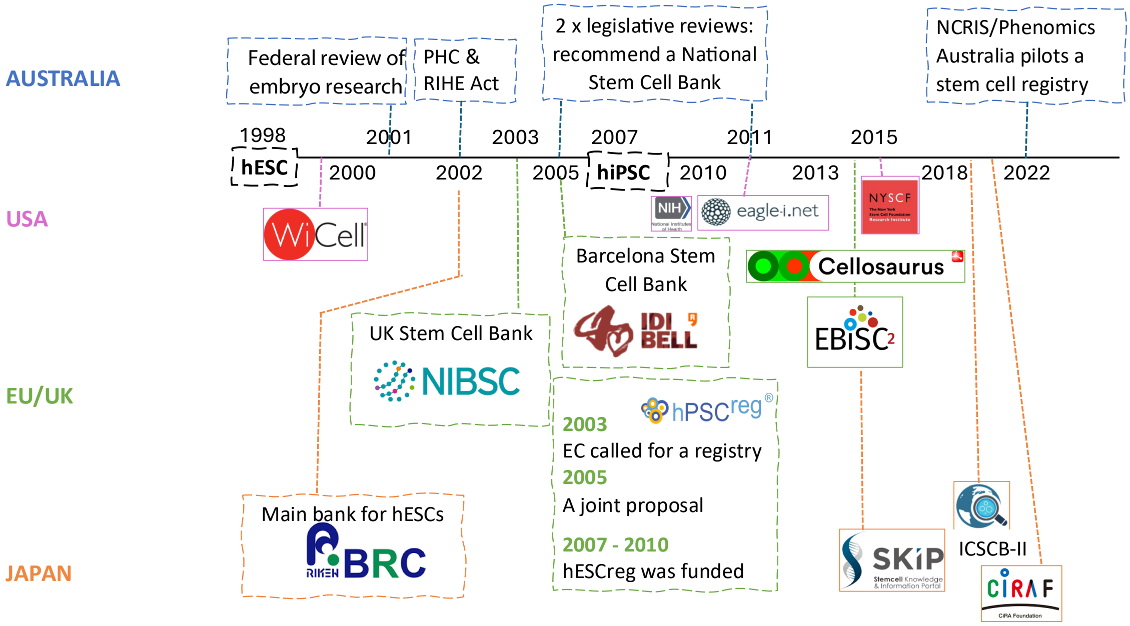


**Figure 2. Timeline of stem cell banks and registries established in Australia, Europe, Japan, and the United States**
PHC, Prohibition of Human Cloning Act; RIHE: Research Involving Human Embryos Act.

supporting infrastructure (National Health and Medical Research, 2019).

The second wave accompanied the expansion of hiPSC technologies, shifting focus on the creation of registries and integrated databases such as hPSCreg, Eagle-i, SKIP, Cellosaurus, and ICSCB. In Australia, a 2011 survey indicated a preference among researchers for a national registry over a bank (ASCC Submission, 2011; Australian Government, 2011), but no investment was made until 2022 when Phenomics Australia funded a pilot registry—Australia's first coordinated effort in this space. Consequently, despite its strong research output, Australia lags by more than 15 years in infrastructure development.

## DISCUSSION

Pluripotent stem cell lines are an important research resource and are widely adopted as models of human biology or human disease. As the field rapidly expands and generates increasing volumes of cell lines and associated (meta)data, we set out to examine whether it was keeping pace with digital best practices. In particular, we examined whether the central information about how a stem cell line was made was routinely captured by stem cell banks and registries. We also assessed how open these stem cell digital infrastructures were relative to FAIR data principles. Overall, this study found that data sharing in the stem cell field has become increasingly aligned with open science principles, reflected in substantial global investment in infrastructures to share stem cell lines and their (meta)data. Of the 21,674 hPSC lines with a digital footprint identified in our study, 11,675 were cited in publications, and 11% of these had been used in multiple studies, indicating utility beyond their initial derivation.

Regional stem cell digital infrastructures in Europe, the US, and Japan collectively account for the majority of hPSC lines indexed in Cellosaurus, highlighting the critical role of coordinated, regionally led efforts in supporting FAIR data capture. Pluripotent stem cell lines from countries like Australia, which currently lacks regional digital infrastructure, were substantially underrepresented in Cellosaurus. Nevertheless, we identified foundational gaps that need to be urgently addressed across these resources to support the findability, accessibility, and interoperability of information held within these resources. Notably, the field faces limited adoption of PIDs by individual databases, mismatched and often unilateral data-sharing controls imposed by infrastructure developers that hinder automated data exchange, and a lack of community-agreed standards for database fields and stem cell terminology. These issues prevent users from finding relevant information, result in duplication, and create confusion around cell line data. We expect that such challenges contribute to a bias against reusing cell lines across studies. We also noticed that only a small number of well-documented cell lines are widely shared, leading to the publication

and citation biases described in this study. Certainly, we acknowledge the contribution of registries and stem cell banks to FAIR and open science principles, which was evidenced by the patterns of registration and banking among cell lines reused across multiple studies. Further addressing foundational issues—such as standardization, governance, and shared practices—is essential to fully realize the potential of applying FAIR principles to the information (metadata) about the establishment of a stem cell line.

A major barrier to interoperability across infrastructures is the limited adoption of PIDs, which are essential for ensuring the digital identity of a cell line and enabling data linkage across platforms (Wells et al., 2024). The use of cell line PIDs could be considered equivalent to DOIs for publications, or barcodes at the supermarket. The PID does not replace cell line names but ensures that lines with similar names can be distinguished from one another. Despite the introduction of PIDs over a decade ago (Luong et al., 2011) and their incorporation into stem cell infrastructures like hPSCreg (Kurtz et al., 2018) and Cellosaurus (Bandrowski et al., 2015), PIDs remain underutilized. This study found that 15 out of 20 investigated infrastructures do not implement PIDs in their websites—a finding consistent with a recent interview study in Australia, which revealed that PIDs are rarely used in researchers' practice (Hu et al., 2024). There have been growing calls recently for the wider adoption of PIDs and increased attention to hPSC registration within the stem cell research community (Hu et al., 2024; Luong et al., 2011; Wells et al., 2024), with their use explicitly recommended in the ISSCR's 2023 guidelines (Ludwig et al., 2023). Further addressing this issue will require continued policy requirements, community support, and funding incentives to advance FAIR data implementation.

To bridge the gap between current and best practices in the stem cell field, aligning digital infrastructures with international standards—such as those established by the ISSCR—is essential. Their 2023 guidelines provide comprehensive recommendations for reporting hPSC data and metadata—including cell line provenance, donor attributes, genomics characterization, and evidence of pluripotency (ISSCR, 2023)—which can serve as a blueprint for what should be registered within digital infrastructures. Aligning data standards with both community needs and established norms will enhance interoperability and facilitate cross-infrastructure data sharing or even cell line sharing. Currently, research remains heavily reliant on a small number of early derived cell lines, particularly those derived from the Yamanaka and Thomson laboratories. For instance, WiCell reported in 2018 that the original five lines from Thomson's lab (H1, H7, H9, H13, and H14) had been distributed 5,200 times to 2,350 independent principal investigators across 820 institutions in 45 countries (Ludwig et al., 2018). However, this study further highlights that most other hPSC lines remain underused or under-cited, limiting the diversity of biological models in research. The Equity Working Group within the Human Cell Atlas, along with many other initiatives, has emphasized the importance of including cell lines derived from diverse populations and environments to better reflect global variability (Amit et al., 2024). Improving the FAIRness of data and metadata for a broader range of cell lines could ultimately support more inclusive, representative, and equitable stem cell research.

Therefore, we advocate for further community-led changes to align valuable local infrastructure with the broader vision of globally open and FAIR research. Specifically, we call for wider adoption of PIDs, more open sharing of cell line metadata with easily searchable web interfaces, and greater consideration of user experience to simplify the registration process within existing data infrastructures. We also support a distributed model in which countries or regions establish centralized nodes of stem cell data infrastructure where they do not yet exist. Additionally, improved communication between data infrastructures is essential to develop shared standards and enable cross-platform data sharing, which can contribute to FAIRness on a global scale. Together, these actions can serve as a strong foundation for change, as described in the research culture change model (Shaw et al., 2022). Over time, such a foundation could help foster upward communication around the need for incentives—from funders, institutions, and journals (Santos et al., 2025) or even through policy changes—which will be crucial in driving broader adoption. In these ways, we can move toward a more transparent, FAIR, and equitable stem cell research ecosystem—one that reduces the risk of unnecessary costs, duplication, or fragmentation arising from uncoordinated infrastructure development that may not align with evolving research needs.

## Limitations of the study

This study encountered a dilemma in FAIR evaluation, which is that data must already possess a certain level of FAIRness to be findable and accessible. This study focused on metadata documented within stem cell infrastructures, as these sources offer standardized and accessible information. However, this approach inherently favors cell lines that are already somewhat FAIR—particularly in terms of findability and accessibility. In contrast, metadata reported only in publications are difficult to capture due to the lack of standardized reporting, requiring extensive manual curation for comprehensive coverage. Even more challenging are metadata stored in non-public platforms, which remain invisible and excluded from evaluation due to their limited discoverability.

The following analysis using Cellosaurus is limited to the data curated within the database. Although Cellosaurus integrates data from various sources—including publications, other databases, websites, and individual submissions—there are inherent limitations in findability and accessibility across these inputs. For example, some cell lines are only mentioned in supplemental information, posing challenges in curation. Furthermore, it may also overlook cell lines documented outside its integrated sources as summarized in Figures 1B and 1D.

## METHODS

### Infrastructure identification and data collection

Stem cell data infrastructures were initially identified from the ICSCB database (Chen et al., 2021) and then expanded through hPSCreg, related websites, and the literature.

Cellosaurus v.49.0 was downloaded as "*cellosaurus.txt*" from the file transfer protocol (FTP) server (https://ftp.expasy.org/databases/cellosaurus/) and filtered for hPSCs using two criteria: (1) cell type categorized as "Embryonic stem cell" or "Induced pluripotent stem cell" (field "CA") and (2) species listed as *Homo sapiens* (OX = NCBI_TaxID = 9606). This yielded 21,674 hPSC lines.

ICSCB data were downloaded on September 25, 2024, from the search result page (https://icscb.stemcellinformatics.org/), with all data sources selected. All records sourced from hPSCreg were retained, including those missing specific stem_cell_type values, as all hPSCreg lines are assumed to be hPSCs. The remaining ICSCB records were filtered to include only those with the following types: "ES Cell," "Human iPS Cell Lines," "Induced Pluripotent Stem Cell Line," and "iPS Cell." This yielded 16,471 hPSC records. After removing nine duplicates, 16,462 unique hPSC entries remained.

### Data integration and analysis

#### *Database integration*

Cross-referencing between Cellosaurus and ICSCB was performed using the "DR" field in Cellosaurus and the "_cellid" field in ICSCB.

#### *Registration, banking, and publication status*

The "RX" and "DR" fields in Cellosaurus were used to determine publication status, registry inclusion, and banking status. A total of 15 registries and banks were identified, and their country of origin was established through manual review.

#### *Field documentation and completion*

From 17 main and 24 subfields under "CC," we selected 13 main and 15 subfields based on relevance and calculated their completion rates (Table 4).

#### *Country assignment and inference*

Since 35% (7,525/21,674) of Cellosaurus hPSC lines lacked country data in the "From" field, country of origin was inferred using multiple strategies: (1) known generation country via registry entries; (2) bank location if the line was banked; and (3) for ICSCB records, institution-level data were used with ChatGPT-3.5 (accessed in October 2024) assistance to infer country names. This recovered data for 2,805 additional lines across various banks and registries.

#### *Citation exploration*

PubMed IDs from Cellosaurus "RX" fields were used to retrieve metadata, citation counts, and abstracts via the Scopus API using the Pybliometrics Python library (Rose and Kitchin, 2019). Total citations per cell line were calculated by summing citations from all linked publications.

#### *Infrastructure timelines*

To understand regional infrastructure development, we conducted a qualitative review of the literature, government reports, and official websites from the US, Europe, Japan, and Australia. The Wayback Machine (https://web.archive.org/ was used to access archived web content when needed.

#### *Statistics*

Publication and citation distributions were assessed using skewness and kurtosis from scipy.stats module. Skewness values between ±0.5–1 indicate moderate skew; values beyond ±1 indicate high skew. A skewness value <0 indicates a left-skewed distribution, whereas a value >0 indicates a right-skewed distribution. Kurtosis >3 denotes a leptokurtic (peaked, heavy-tailed) distribution, while <3 indicates a platykurtic (flat, light-tailed) one. Mean and median were also calculated to assess central tendency.

To test the correlation between the year of publication and citation count, citations were transformed by adding 1 (to avoid log(0)) and then $\log_{10}$-transformed using the NumPy package. An ordinary least square regression was performed, with $p < 0.05$ considered statistically significant.

## RESOURCE AVAILABILITY

### Lead contact

Further information and requests for resources should be directed to and will be fulfilled by the lead contact, Christine A. Wells (wells.c@unimelb.edu.au).

### Materials availability

This study did not generate new unique reagents.

### Data and code availability

- All original code has been deposited at GitHub and is publicly available at https://github.com/wellslab as of the date of publication. Any additional information required to

reanalyze the data reported in this paper is available from the lead contact upon request.

- This paper analyses existing, publicly available data, accessible at URLs shown in Table 2. All data processing and analysis were conducted in Python within the Google Collab environment. During the preparation of this work, the first author used ChatGPT to improve readability. After using this tool, the author reviewed and edited the content as needed and took full responsibility for the content of the publication.


## ACKNOWLEDGMENTS

The authors gratefully acknowledge discussions with Amos Bairoch from Cellosaurus and the EAOR team.


## AUTHOR CONTRIBUTIONS

Conceptualization, M.H., C.A.W., and R.A.A. Data curation, M.H. Formal analysis, M.H. Funding acquisition, C.A.W. and R.A.A. Investigation, C.A.W. and R.A.A. Project administration, C.A.W. and R.A.A. Supervision, C.A.W. and R.A.A. Writing – original draft, M.H. Writing – review and editing, M.H., D.S., C.A.W., and R.A.A.

## DECLARATION OF INTERESTS

C.A.W. is funded to implement the Australian stem cell registry.

## SUPPLEMENTAL INFORMATION

Supplemental information can be found online at https://doi.org/10.1016/j.stemcr.2025.102644.


Received: April 11, 2025
Revised: August 21, 2025
Accepted: August 22, 2025
Published: September 25, 2025


## REFERENCES


Amit, I., Ardlie, K., Arzuaga, F., Awandare, G., Bader, G., Bernier, A., Carninci, P., Donnelly, S., Eils, R., Forrest, A.R.R., et al. (2024). The commitment of the human cell atlas to humanity. Nat. Commun. *15*, 10019. https://doi.org/10.1038/s41467-024-54306-x.

Australian Government (2005). Legislation review: Prohibition of Human Cloning Act 2002, Research Involving Human Embryos Act 2002 (Canberra: Dept. of Health and Ageing).

Australian Government (2011). Legislation review: Prohibition of Human Cloning for Reproduction Act 2002 and the Research Involving Human Embryos Act 2002. 96.

Australian Research Data Commons. (2018). Home. https://ardc.edu.au/.

Australian Research Data Commons (2024). Australian National Persistent Identifier (PID) Strategy. https://doi.org/10.5281/ZENODO.10656275.

Bairoch, A. (2018). The Cellosaurus, a Cell-Line Knowledge Resource. J. Biomol. Tech. *29*, 25–38. https://doi.org/10.7171/jbt.18-2902-002.

Bandrowski, A., Brush, M., Grethe, J.S., Haendel, M.A., Kennedy, D. N., Hill, S., Hof, P.R., Martone, M.E., Pols, M., Tan, S., et al. (2015). The Resource Identification Initiative: A cultural shift in publishing. F1000Res. *4*, 134. https://doi.org/10.12688/f1000research.6555.2.

Baysoy, A., Bai, Z., Satija, R., and Fan, R. (2023). The technological landscape and applications of single-cell multi-omics. Nat. Rev. Mol. Cell Biol. *24*, 695–713. https://doi.org/10.1038/s41580-023-00615-w.

Canet-Avilés, R. (2025). Memorandum for CIRM iPSC Repository Closure [Memorandum]. https://www.cirm.ca.gov/wp-content/uploads/2025/05/Memo-CIRM-iPSC-Bank-Closure-0520-2025.pdf.

Chen, Y., Sakurai, K., Maeda, S., Masui, T., Okano, H., Dewender, J., Seltmann, S., Kurtz, A., Masuya, H., Nakamura, Y., et al. (2021). Integrated Collection of Stem Cell Bank Data, a Data Portal for Standardized Stem Cell Information. Stem Cell Rep. *16*, 997–1005. https://doi.org/10.1016/j.stemcr.2021.02.014.

European Commission (2015). European Open Science Cloud. https://research-and-innovation.ec.europa.eu/strategy/strategy-research-and-innovation/our-digital-future/open-science/european-open-science-cloud-eosc_en.

European Commission (2016). H2020 Programme. In Guidelines on FAIR Data Management in Horizon 2020, No. Version 3.0 (European Commission, DirectorateGeneral for Research&Innovation).

Fecher, B., and Friesike, S. (2014). Open Science: One Term, Five Schools of Thought. In Opening Science: The Evolving Guide on How the Internet Is Changing Research, Collaboration and Scholarly Publishing, S. Bartling and S. Friesike, eds. (Springer International Publishing), pp. 17–47.

GO, FAIR Initiative (2016). The FAIR Principles. *Standards for Human Stem Cell Use in Research*. https://static1.squarespace.com/static/611faaa8fee682525ee16489/t/657b6db5d4384322efa07218/1702587832597/ISSCR_Standards_Dec23_Update.pdf.

Hu, M., Santos, D., Lopes, E., Nicol, D., Kurtz, A., Mah, N., Muller, S., Ankeny, R.A., and Wells, C.A. (2024). Australian researchers' perceptions and experiences with stem cell registration. Stem Cell Res. *79*, 103482. https://doi.org/10.1016/j.scr.2024.103482.

ISSCR (2023). Standards for Human Stem Cell Use in Research.

International Society for Stem Cell Research (ISSCR). https://static1.squarespace.com/static/611faaa8fee682525ee16489/t/657b6db5d4384322efa07218/1702587832597/ISSCR_Standards_Dec23_Update.pdf.

Jacobsen, A., De Miranda Azevedo, R., Juty, N., Batista, D., Coles, S., Cornet, R., Courtot, M., Crosas, M., Dumontier, M., Evelo, C.T., et al. (2020). FAIR Principles: Interpretations and Implementation Considerations. Data Intell. *2*, 10–29. https://doi.org/10.1162/dint_r_00024.

Kim, J.-H., Jo, H.-Y., Ha, H.-Y., and Kim, Y.-O. (2021). Korea National Stem Cell Bank. Stem Cell Res. *53*, 102270. https://doi.org/10.1016/j.scr.2021.102270.

Kurtz, A., Seltmann, S., Bairoch, A., Bittner, M.-S., Bruce, K., Capes-Davis, A., Clarke, L., Crook, J.M., Daheron, L., Dewender, J., et al. (2018). A Standard Nomenclature for Referencing and

Authentication of Pluripotent Stem Cells. Stem Cell Rep. *10*, 1–6. https://doi.org/10.1016/j.stemcr.2017.12.002.

Ludwig, T.E., Kujak, A., Rauti, A., Andrzejewski, S., Langbehn, S., Mayfield, J., Fuller, J., Yashiro, Y., Hara, Y., and Bhattacharyya, A. (2018). 20 Years of Human Pluripotent Stem Cell Research: It All Started with Five Lines. Cell Stem Cell *23*, 644–648. https://doi.org/10.1016/j.stem.2018.10.009.

Ludwig, T.E., Andrews, P.W., Barbaric, I., Benvenisty, N., Bhattacharyya, A., Crook, J.M., Daheron, L.M., Draper, J.S., Healy, L.E., Huch, M., et al. (2023). ISSCR standards for the use of human stem cells in basic research. Stem Cell Rep. *18*, 1744–1752. https://doi.org/10.1016/j.stemcr.2023.08.003.

Luong, M.X., Auerbach, J., Crook, J.M., Daheron, L., Hei, D., Lomax, G., Loring, J.F., Ludwig, T., Schlaeger, T.M., Smith, K.P., et al. (2011). A Call for Standardized Naming and Reporting of Human ESC and iPSC Lines. Cell Stem Cell *8*, 357–359. https://doi.org/10.1016/j.stem.2011.03.002.

Martins de Oliveira, M.L., Tura, B.R., Meira Leite, M., Melo dos Santos, E.J., Pôrto, L.C., Pereira, L.V., and Campos de Carvalho, A.C. (2023). Creating an HLA-homozygous iPS cell bank for the Brazilian population: Challenges and opportunities. Stem Cell Rep. *18*, 1905–1912. https://doi.org/10.1016/j.stemcr.2023.09.001.

National Institute of Health (2023). Final NIH Policy for Data Management and Sharing. https://grants.nih.gov/grants/guide/notice-files/NOT-OD-21-013.html.

Rose, M.E., and Kitchin, J.R. (2019). pybliometrics: Scriptable bibliometrics using a Python interface to Scopus. SoftwareX *10*, 100263. https://doi.org/10.1016/j.softx.2019.100263.

National Health and Medical Research Council. (2019). *NHMRC Embryo Research Licensing Committee: Report to the Parliament of Australia for the period 1 March 2019 to 31 August 2019*. Canberra: National Health and Medical Research Council.

Royan institute (2020). Royan Stem Cell Bank. https://web.archive.org/web/20201001144644/http:/www.royaninstitute.org/cmsen/index.php?option=com_content&task=view&id=205&Itemid=40.

Santos, D., Hu, M., Lopes McInnes, E., Wells, C., Leach, J., Nicol, D., and Ankeny, R.A. (2025). Commoning contingent resources: Constructing an Australian stem cell registry. New Genet. Soc. *44*, e2491997. https://doi.org/10.1080/14636778.2025.2491997.

Seltmann, S., Lekschas, F., Müller, R., Stachelscheid, H., Bittner, M.-S., Zhang, W., Kidane, L., Seriola, A., Veiga, A., Stacey, G., and Kurtz, A. (2016). hPSCreg—the human pluripotent stem cell registry. Nucleic Acids Res. *44*, D757–D763. https://doi.org/10.1093/nar/gkv963.

Shaw, L.C., Errington, T.M., and Mellor, D.T. (2022). Toward Open Science: Contributing to Research Culture Change. Sci. Ed. *45*, 14–17. https://doi.org/10.36591/SE-D-4501-14.

Staerk, J., Dawlaty, M.M., Gao, Q., Maetzel, D., Hanna, J., Sommer, C.A., Mostoslavsky, G., and Jaenisch, R. (2010). Reprogramming of Human Peripheral Blood Cells to Induced Pluripotent Stem Cells. Cell Stem Cell *7*, 20–24. https://doi.org/10.1016/j.stem.2010.06.002.

Takahashi, K., Tanabe, K., Ohnuki, M., Narita, M., Ichisaka, T., Tomoda, K., Yamanaka, S., Tomoda, K., and Yamanaka, S. (2007). Induction of Pluripotent Stem Cells from Adult Human Fibroblasts by Defined Factors. Cell *131*, 861–872. https://doi.org/10.1016/j.cell.2007.11.019.

Wang, M., and Savard, D. (2023). The FAIR Principles and Research Data Management. In Research Data Management in the Canadian Context, K. Thompson, E. Hill, E. Carlisle-Johnston, D. Dennie, and É. Fortin, eds. (Western University, Western Libraries).

Wang, L., Ding, J., Stacey, G.N., and Hao, J. (2021). The Chinese National Stem Cell Resource Center. Stem Cell Res. *50*, 101985. https://doi.org/10.1016/j.scr.2020.101985.

Wells, C.A., Guhr, A., Bairoch, A., Chen, Y., Hu, M., Löser, P., Ludwig, T.E., Mah, N., Mueller, S.C., Seiler Wulczyn, A.E.M., et al. (2024). Guidelines for managing and using the digital phenotypes of pluripotent stem cell lines. Stem Cell Rep. *19*, 1369–1378. https://doi.org/10.1016/j.stemcr.2024.08.009.

Wilkinson, M.D., Dumontier, M., Aalbersberg, I.J., Appleton, G., Axton, M., Baak, A., Blomberg, N., Boiten, J.-W., da Silva Santos, L.B., Bourne, P.E., et al. (2016). The FAIR Guiding Principles for scientific data management and stewardship. Sci. Data *3*, 160018. https://doi.org/10.1038/sdata.2016.18.

Yu, J., Vodyanik, M.A., Smuga-Otto, K., Antosiewicz-Bourget, J., Frane, J.L., Tian, S., Nie, J., Jonsdottir, G.A., Ruotti, V., Stewart, R., et al. (2007). Induced Pluripotent Stem Cell Lines Derived from Human Somatic Cells. Science *318*, 1917–1920.

Australian Stem Cell Centre. (2011). Submission to 2010 Legislation Review (Submission No. 231; pp. 16–17). https://web.archive.org/web/20110406022440/https://legislationreview.nhmrc.gov.au/sites/default/files/submissions/231%20-%20ASCC%20Submission_2010%20Review%5B1%5D.pdf.

MEXT (2002). The Ministry of Education, Culture, Sports, Science and Technology (MEXT). 2002. National BioResource Project. https://nbrp.jp/en/about-en/.

**Stem Cell Reports, Volume *20***

## Supplemental Information

# How FAIR is metadata for human pluripotent stem cells?

**Mengqi Hu, Rachel A. Ankeny, Dan Santos, and Christine A. Wells**

Hu et al Stem Cell Reports How FAIR is metadata for human pluripotent stem cells?

Supplementary Files Index

**Figure S1**. Effect of Time (Publication Year) on Citation Count of Cell Lines. Citations of published cell lines are shown in a dot plot, with the Y-axis representing publication year and the X-axis representing citation count. Citation counts were log-transformed (log10 + 1) to ensure all values are positive. A linear regression line (red) was fitted using Ordinary Least Squares (OLS), with model coefficients shown at the top of the figure. The association between publication year and the amount of citation is statistically significant ($P < 0.0001$), with earlier-published cell lines tending to have more citations

**Figure S2**. hPSC across Jurisdictions Identified in Cellosaurus. The X-axis represents 28 jurisdictions identified from the Cellosaurus database, and the Y-axis shows the number of hPSC lines associated with each jurisdiction

**Figure S3**. Data sources for Australian hPSCs documented in Cellosaurus. Venn diagram shows the three key data sources—hPSCreg, NIHhESC, and SKIP—used by Cellosaurus to capture hPSC lines originating from Australia.

**Table S1**. Detailed Framework for FAIR Evaluation of hPSC Data Infrastructures. The left column detailed 15 guiding FAIR principles from GO FAIR (2025) and Wilkinson et al. (2016). The corresponding "Assessment Criteria" column on the right presents our translated evaluation framework for stem cell data infrastructures. Principles shown shaded in grey represent those that are challenging to assess or not directly applicable to stem cell infrastructures

**Table S2**. Integration of ICSCB and Cellosaurus Data Fields Illustrated by H9 Records. (See in the excel file)

**Table S3**. Completeness of Data Fields in Cellosaurus. This table presents 15 main and 13 subfields from Cellosaurus that are relevant to stem cell data, organized by ISSCR information categories. Completeness rates are calculated for each field and color-coded as follows: green (>90%), yellow (20–90%), and red (<20%).

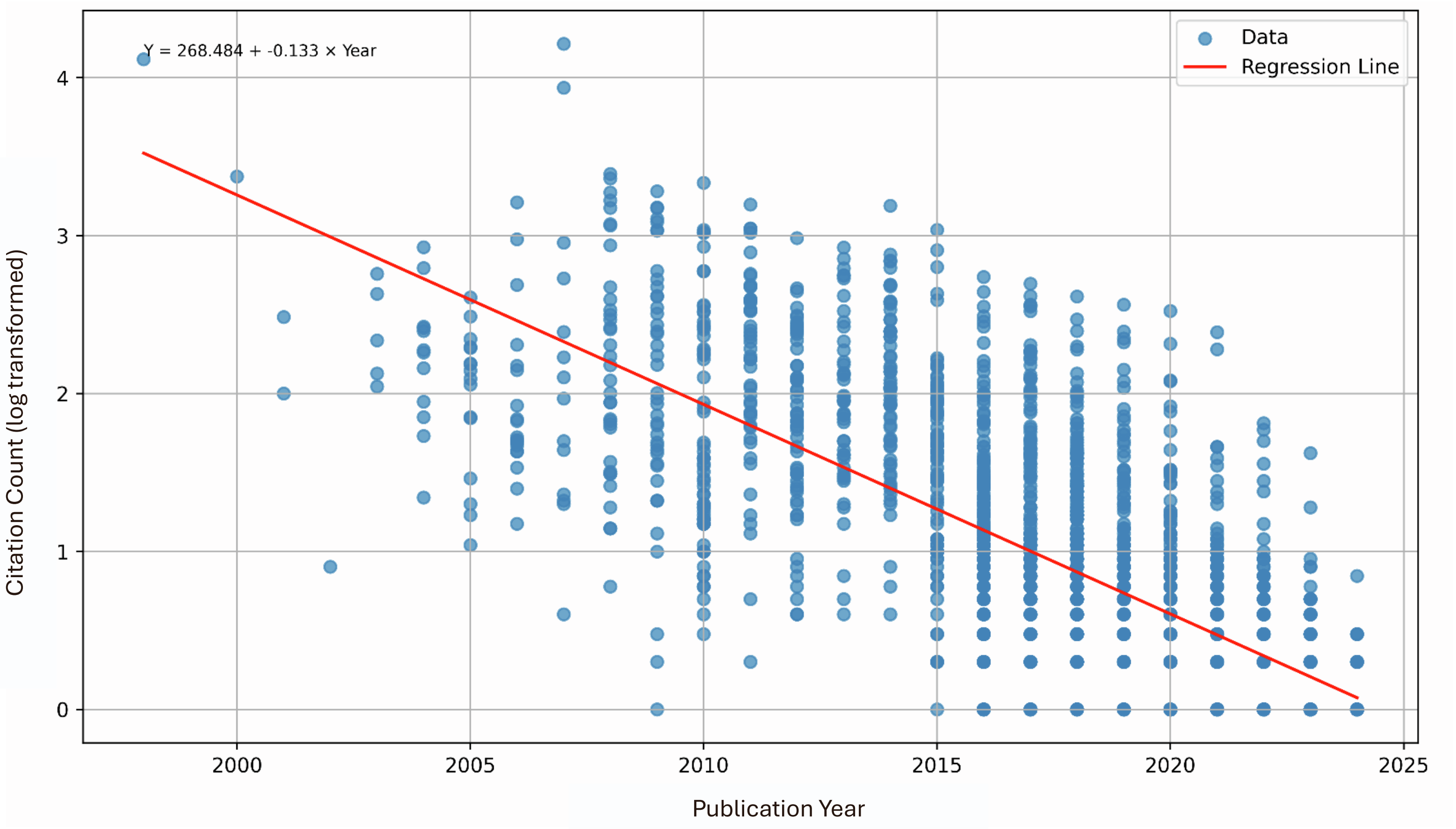


**Figure S1. Effect of Time (Publication Year) on Citation Count of Cell Lines.** Citations of published cell lines are shown in a dot plot, with the Y-axis representing publication year and the X-axis representing citation count. Citation counts were log-transformed ($\log_{10} + 1$) to ensure all values are positive. A linear regression line (red) was fitted using Ordinary Least Squares (OLS), with model coefficients shown at the top of the figure. The association between publication year and the amount of citation is statistically significant ($P < 0.0001$), with earlier-published cell lines tending to have more citations.

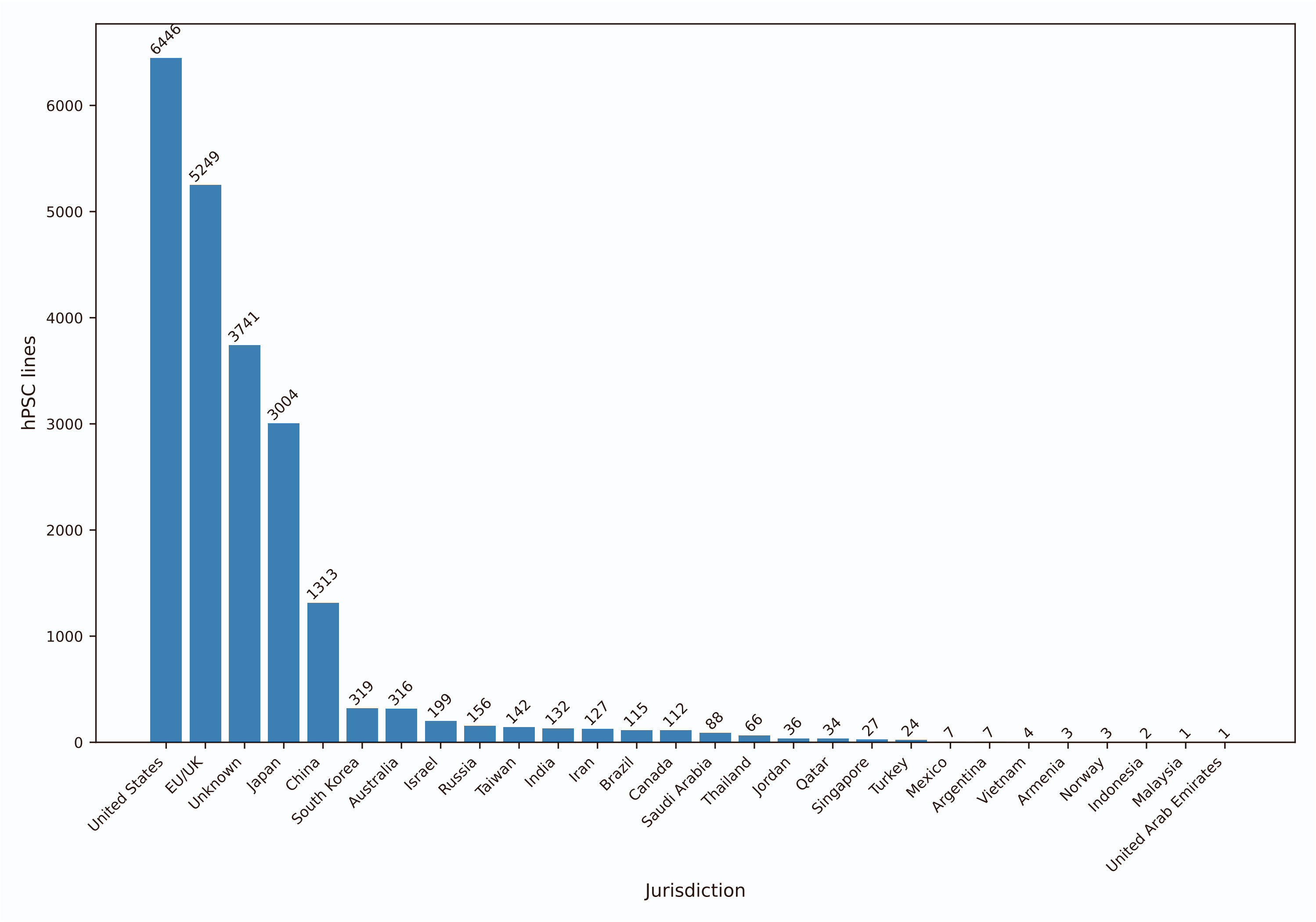


**Figure S2. hPSC across Jurisdictions Identified in Cellosaurus.** The X-axis represents 28 jurisdictions identified from the Cellosaurus database, and the Y-axis shows the number of hPSC lines associated with each jurisdiction.

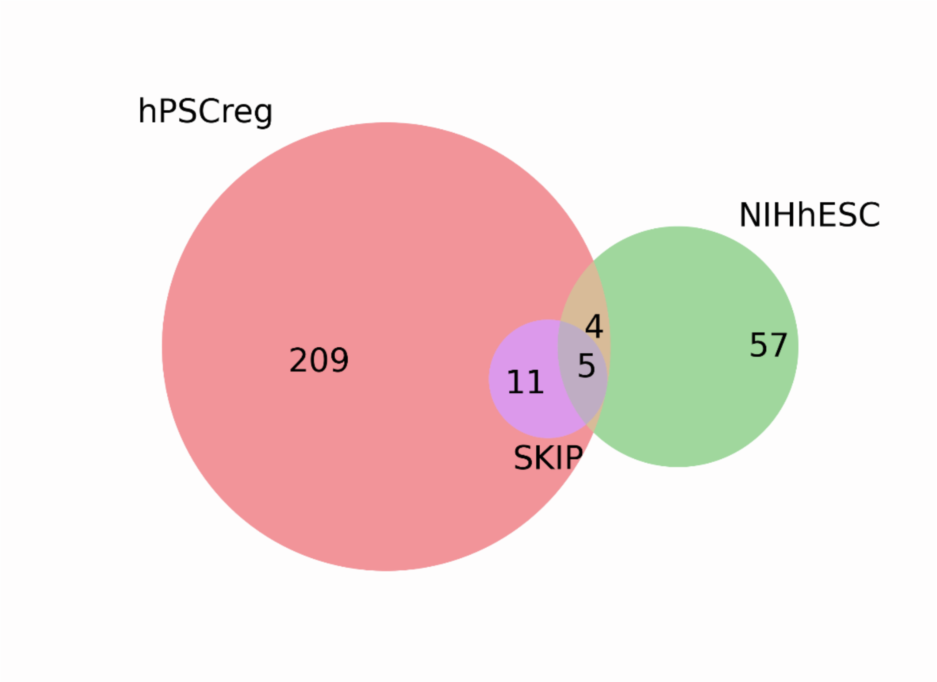


**Figure S3. Data sources for Australian hPSCs documented in Cellosaurus.** Venn diagram shows the three key data sources—hPSCreg, NIHhESC, and SKIP—used by Cellosaurus to capture hPSC lines originating from Australia.

**Table S1. Detailed Framework for FAIR Evaluation of hPSC Data Infrastructures.** The left column detailed 15 guiding FAIR principles from GO FAIR (2025) and Wilkinson et al. (2016). The corresponding "Assessment Criteria" column on the right presents our translated evaluation framework for stem cell data infrastructures. Principles shown shaded in grey represent those that are challenging to assess or not directly applicable to stem cell infrastructures.

| FAIR Guiding Principles (Go Fair, 2025; Wilkinson et al., 2016a) | | | Assessment Criteria |
|---|---|---|---|
| **Findable (F)<br>The first step in (re)using data is to find them. Metadata and data should be easy to find for both humans and computers. Machine-readable metadata are essential for the automatic discovery of datasets and services, so this is an essential component of the FAIRification process.** | F1. (Meta)data are assigned a globally unique and persistent identifier. | | Is a globally unique and persistent identifier assigned to the cell line metadata in this infrastructure? |
| | F2. Data are described with rich metadata (defined by R1 below). | | (See R1) |
| | F3. Metadata clearly and explicitly includes the identifier of the data they describe. | | Are identifiers assigned to the cell lines described in the metadata?<br>Does the metadata in this infrastructure cross-link to other (meta)data? |
| | F4. (Meta)data are registered or indexed in a searchable resource | | Is metadata searchable within the infrastructure? |
| **Accessible (A)<br>Once the user finds the required data, she/he/they need to know how they can be accessed, possibly including authentication and authorisation.** | A1. (Meta)data are retrievable by their identifier using a standardised communications protocol. | A1.1 The protocol is open, free, and universally implementable. | Is metadata accessible from the infrastructure?<br>Can the metadata be downloaded in a machine-readable format? |
| | | A1.2 The protocol allows for an authentication and authorisation procedure, where necessary. | |
| | A2. Metadata is accessible, even when the data are no longer available | | Is the metadata still accessible when the infrastructure is retired? |
| **Interoperable (I)<br>The data usually needs to be integrated with other data. In addition, the data needs to interoperate with applications or workflows for analysis, storage, and processing.** | I1. (Meta)data use a formal, accessible, shared, and broadly applicable language for knowledge representation. | | How does the use of PID affect metadata integration and comparison across other platforms?<br>How does data structure affect integration and comparison across other platforms? |
| | I2. (Meta)data use vocabularies that follow FAIR principles. | | How do data standards impact data integration and comparison across other platforms? |
| | I3. (Meta)data include qualified references to other (meta)data. | | - |
| **Reusable (R)<br>The ultimate goal of FAIR is to optimise the reuse of data. To achieve this, metadata and data should be well-described so that they can be replicated and/or combined in different settings.** | R1. (Meta)data are richly described with a plurality of accurate and relevant attributes. | R1.1. (Meta)data are released with a clear and accessible data usage license | - |
| | | R1.2. (Meta)data are associated with detailed provenance. | Does the metadata include information about the provenance of cell lines? |
| | | R1.3. (Meta)data meet domain-relevant community standards. | Does the metadata contain a lot of missing values?<br>Does the metadata in this infrastructure comply with ISSCR guidelines? |

**Table S2. Integration of ICSCB and Cellosaurus Data Fields Illustrated by H9 Records.** (See in the excel file)

**Table S3. Completeness of Data Fields in Cellosaurus.** This table presents 15 main and 13 subfields from Cellosaurus that are relevant to stem cell data, organized by ISSCR information categories. Completeness rates are calculated for each field and color-coded as follows: green (>90%), yellow (20–90%), and red (<20%).

| ISSCR Category | Field Type | Field Name | Explanation | Completeness Rate |
|---|---|---|---|---|
| Basic Characteristics | Main | AC | Cellosaurus accession number | 100% |
| | Main | ID | Cell line original ID | 100% |
| | Main | DT | Date of creation, last updated, version | 100% |
| | Main | DR | Cross-references to external databases | 100% |
| | Main | CA | Type of stem cell (iPSC/ESC) | 100% |
| | Main | SY | List of synonyms | 71% |
| | Subfield | From | Institution of origin | 65% |
| | Main | RX | References IDs for publication or patent | 54% |
| | Main | OI | Derived from the same individual | 31% |
| | Main | HI | Parental or descendant cell lines | 25% |
| | Subfield | Discontinued | Not available in a bank | 14% |
| | Subfield | Part of | Part of a bank, study or consortium | 10% |
| | Main | ST | Short tandem repeat (STR) profile | 6% |
| | Subfield | Registration | Registered or banked infrastructures | 4% |
| | Subfield | Group | Clinical-grade or patented status | 0% |
| | Subfield | Problematic cell line | Misidentified, contaminated, or from a retracted paper | 0% |
| Stem Cell-Based Model Systems | Main | SX | Donor’s sex | 97% |
| | Main | AG | Donor’s age at sampling | 93% |
| | Subfield | Derived from site | Tissue sites | 91% |
| | Subfield | Cell type | Original tissue type | 59% |
| | Main | DI | Associated diseases | 48% |
| | Subfield | Population | Ethnicity | 45% |
| Genomic Characterization | Subfield | Sequence variation | Mutation details | 23% |
| | Subfield | Characteristics | Genetic modificatiosn | 12% |
| | Subfield | Omics | Types of omics analysis performed | 12% |
| | Subfield | Knockout cell | Gene knockout information | 2% |
| | Subfield | HLA typing | HLA typing data | 2% |
| | Subfield | Karyotypic information | Karyotypes | 0% |
